# Homebrew: An economical and sensitive glassmilk-based nucleic-acid extraction method for SARS-CoV-2 diagnostics

**DOI:** 10.1016/j.crmeth.2022.100186

**Published:** 2022-03-03

**Authors:** Robert Page, Edward Scourfield, Mattia Ficarelli, Stuart W. McKellar, Kwok Leung Lee, Thomas J.A. Maguire, Clement Bouton, Maria Jose Lista, Stuart J.D. Neil, Michael H. Malim, Mark Zuckerman, Hannah E. Mischo, Rocio T. Martinez-Nunez

**Affiliations:** 1 ImmunoEngineering Lab, School of Cancer and Pharmaceutical Sciences, King's College London, Guy's Cancer Centre, Great Maze Pond, London SE1 9RT, UK; 2 Department Infectious Diseases, School of Immunology and Microbial Sciences, Guy’s Campus, King’s College London, London SE1 9RT, UK; 3 South London Specialist Virology Centre, King’s College Hospital London, London, UK

**Keywords:** SARS-CoV-2, COVID-19, low-cost RNA extraction, qPCR diagnostics, molecular diagnostics

## Abstract

Management of COVID-19 and other epidemics requires large-scale diagnostic testing. The gold standard for severe acute respiratory syndrome coronavirus 2 (SARS-CoV-2) infection remains reverse transcription quantitative PCR (qRT-PCR) analysis, which detects viral RNA more sensitively than any other method. However, the resource use and supply-chain requirements of RT-PCR have continued to challenge diagnostic laboratories worldwide. Here, we establish and characterize a low-cost method to detect SARS-CoV-2 in clinical combined nose and throat swabs, allowing for automation in high-throughput settings. This method inactivates virus material with sodium dodecylsulfate (SDS) and uses silicon dioxide as the RNA-binding matrix in combination with sodium chloride (NaCl) and isopropanol. With similar sensitivity for SARS-CoV-2 viral targets but a fraction of time and reagent expenditure compared with commercial kits, our method also enables sample pooling without loss of sensitivity. We suggest that this method will facilitate more economical widespread testing, particularly in resource-limited settings.

## Highlights

• Homebrew is a fast, cheap, validated RNA extraction method for SARS-CoV-2 testing
• Homebrew RNA extraction only requires glassmilk, NaCl, SDS, isopropanol, and ethanol
• Homebrew is scalable and allows for sample pooling without loss of sensitivity

## Motivation

Large-scale diagnostics will be required long term for COVID-19 and future pandemics. Gold-standard PCR requires RNA extraction for increased sensitivity; however, supply chains and costs of commercial kits challenge many laboratories worldwide. We have established a sensitive, low-cost, automatable RNA extraction method in which samples can be pooled. Homebrew employs glassmilk and common laboratory reagents to increase diagnostic capacity in resource-limited settings.

## Graphical abstract

**Figure:**
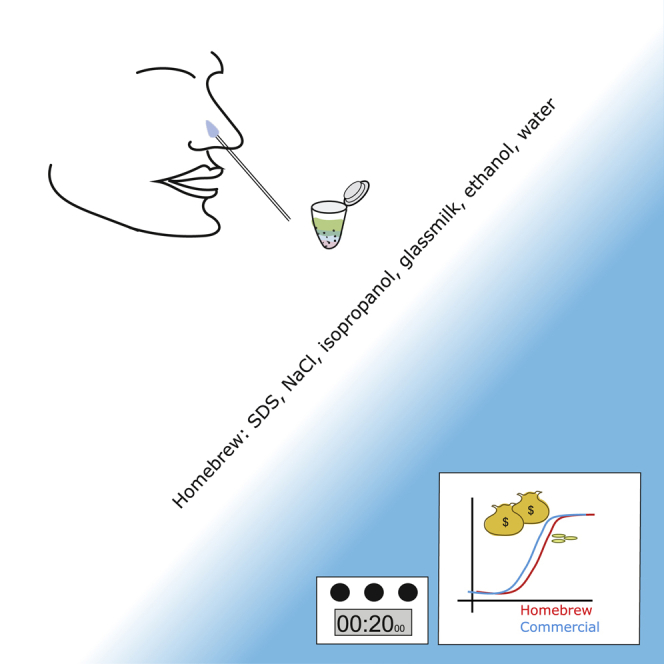

## Teaser

Diagnostics will be required long term for COVID-19 and future pandemics. Page and Scourfield et al. present a sensitive, low-cost, and automatable RNA extraction method for detection of SARS-CoV-2 in clinical samples employing glassmilk and common laboratory reagents to increase diagnostic capacity in resource-limited settings.

## Introduction

As the world approaches the 21^st^ month since COVID-19 was declared a pandemic by the World Health Organization (WHO), global infections are still uncontrolled. Even in countries where combined vaccination and non-pharmacological strategies to contain the virus have decreased hospitalization and death rates, new severe acute respiratory syndrome coronavirus 2 (SARS-CoV-2) variants are causing local outbreaks. Therefore, widespread testing and contact tracing will remain as key tools for control of SARS-CoV-2 for the foreseeable future.

The gold standard for SARS-CoV-2 diagnostics consists of viral RNA extraction from combined nose and throat swab samples followed by qRT-PCR (quantitative reverse transcription and polymerase chain reaction). Standard RNA extraction for SARS-CoV-2 diagnostics is usually carried out with commercial kits, typically based on solid-phase reversible immobilization (SPRI) of nucleic acids. While these kits are optimized to purify high-quality RNA for extremely sensitive downstream applications, in resource-limiting situations, their high cost per sample and requirement for dedicated supply chains and expertise can be a barrier to more widespread testing. Purification-free methods, such as SalivaDirect, hid-RT-PCR, SwabExpress, or others (Smyrlaki et al., 2020; Srivatsan et al., 2021; Vogels et al., 2021), reduce time and costs but compromise in sensitivity and/or require optimization (e.g., depending on sampling buffer). Rapid methods have shown an increase in sensitivity when a rapid purification step is included (Joung et al., 2020). Antigen testing with lateral flow devices provides a fast alternative solution, but such tests only detect peak infectivity with high confidence and are recommended for use in symptomatic individuals (Dinnes et al., 2021; Pickering et al., 2021). These methods are therefore less suited for testing samples containing low viral loads, such as those present at the initial stages of infection, or for population-wide screening of asymptomatic individuals when and where other, more sensitive methods are available.

Prevention of outbreaks therefore requires highly sensitive methods that allow early detection. Thus, to control transmission but also for variant detection, RNA isolation and qRT-PCR testing will continue to be irreplaceable.

While assessing different commercial kits for SARS-CoV-2 (Lista et al., 2021), we encountered bottlenecks in sourcing reagents, also reported elsewhere (Esbin et al., 2020). We sought to establish an economical, scalable, and simplified method of RNA extraction circumventing worldwide shortages in reagents. Here, we describe a method using only NaCl, SDS, isopropanol, and ethanol, present in most laboratories worldwide, for the rapid extraction of SARS-CoV-2-RNA from combined nose and throat swab samples using silica powder (“glassmilk” [GM]) as SPRI matrix. GM-based nucleic acid isolation was originally described in the 1970s (Boyle and Lew, 1995; Vogelstein and Gillespie, 1979), employed later on for sequencing (Dederich et al., 2002) and also SARS-CoV-2 RNA isolation combined with RT-LAMP (Rabe and Cepko, 2020). Developing an adjusted purification method, we established that GM can be replaced with carboxy magnetic beads that allow for automation. We provide a full characterization of the limits of this method and demonstrate that the GM purification method allows for sample pooling without loss of sensitivity, as well as reduced plastic consumption. GM-nucleic acid purification could therefore contribute significantly to resource saving and supply-chain management in a world that will require long-term testing facilities for testing for SARS-CoV2 and other emerging viruses.

## Results

### Establishing the components of a low-cost RNA extraction method

**Figure 1:**
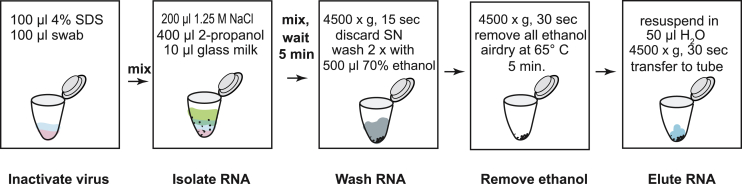
Schema of the five steps of the homebrew method

We set out to establish an alternative RNA extraction method for SARS-CoV-2 testing that would free our setting from supply-chain shortages. Figure 1A summarizes the five optimized steps of our GM method that takes about 15 min per sample to perform (about 20 min per 24 samples). In the following, we describe how we established and stress tested this method using heat-inactivated combined nose and throat swabs in comparison to the gold-standard-rated (Centers for Disease Control and Prevention [CDC]), SPRI-based QIAamp Viral RNA Mini kit (QIAamp hereafter). As combined nose and throat swabs can greatly differ (e.g., viscosity, material, pH, presence of inhibitors, etc.), employing clinical samples when developing new methods, rather than virus-spiked viral transport media (VTM), is paramount to establish reliability, sensitivity, and specificity of new methods.

Nucleic acids are known to reversibly bind silica in the presence of chaotropic salts and non-basic pH conditions (Tan and Yiap, 2009). While most commercial RNA extraction kits employ silica-coated magnetic beads or silica-based columns, we opted to use a suspension of silica particles as a solid matrix for RNA extraction. This suspension, also known as GM, is economic and prepared by simply resuspending HCl-washed silica particles in water. GM particles are large enough to be efficiently pelleted by pulse centrifugation, enabling separation of the solid and liquid phases during extraction.

**Figure 2:**
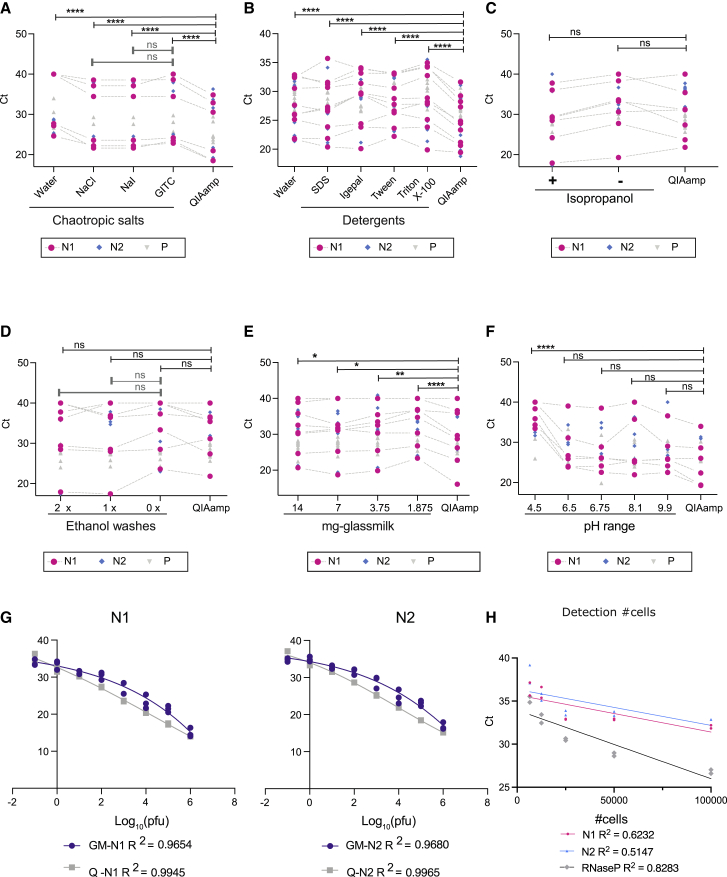
Characterization of minimal components required for RNA extraction (A) Chaotropic salt titration: viral RNA extracted from six combined nose and throat swabs using different chaotropic salts, 2-propanol and 7 mg GM, shows in a one-way ANOVA test significant increase in Ct values compared with the benchmark QIAamp viral RNA mini kit (adjusted p < 0.0001 for water, NaCl, NaI, and guanidium thiocyanate [GITC]). NaCl and NaI had 100% sensitivity, whereas GITC and water lost one and three samples, respectively. ∗∗∗∗ adjusted p < 0.0001. (B) Detergent titration: addition of different detergents prior to RNA extraction from 10 combined nose and throat swabs shows a significant difference to QIAamp (adjusted p one-way ANOVA of <0.0001). All detergents but Tween 20 have been shown to inactivate SARS-CoV-2 at 1% (Patterson et al., 2020). However, the mean difference (ΔCt) compared with the QIAamp viral RNA mini kit differs for each detergent: SDS (−2.579), Igepal (−3.929), Tween (−3.481), Triton (−3.968), and water (−2.637). (C) Isopropanol increases GM binding: one-way ANOVA test shows non-significant differences by adjusted p of ns = 0.4428 (+) and ns = 0.1702 (−) isopropanol. However, the mean difference was compared with QIAamp (ΔCt; +) = 1.104 and (−) = −1.742. (D) Matrix wash with ethanol increases sensitivity. One-way ANOVA test shows no significant differences to the QIAamp viral RNA mini kit. No wash (ns = 0.1971; mean ΔCt = −2.02), 1× wash (ns = 0.9961; mean ΔCt = 0.1935), and 2× washes (ns = 0.9986; mean ΔCt = −0.1353) are shown. (E) GM quantities. ANOVA test with Dunnett’s multiple comparison test versus QIAamp: QIAmp versus 14, adjusted p = 0.0145, QIAmp versus 7 adjusted p = 0.0368, QIAmp versus 3.75 adjusted p = 0.0024 and QIAmp versus 1.875 adjusted p < 0.0001. (F) Effect of pH of the RNA-extraction buffer on detection of viral RNA from six combined nose and throat swabs compared with QIAamp: pH 4.5 (adjusted p < 0.0001), pH 6.5 (ns = 0.3709), pH 6.75 (ns = 0.2187), pH 8.1 (ns = 0.2433), and pH 9 (ns = 0.5434). (G) Level of detection of QIAamp, compared with the homebrew method using GM and 10-fold serial dilutions of SARS CoV-2 in viral transport medium. Ct, cycle threshold; pfus, plaque-forming units. The dilution series was best fit with a non-linear regression. (H) Level of detection of RNase P, N1, and N2 employing homebrew GM depending on cell numbers present in the sample. BEAS-2B cells were prepared at different numbers and spiked with equal amounts of SARS-CoV-2 and assessed using GM. The dilution series was best fit with a linear regression.

Most commercial RNA extraction kits employ guanidinium thiocyanate (GITC) as chaotropic and protein-denaturing salt to induce binding of nucleic acids to silica. However, the global shortage of GITC combined with its toxicity prompted us to test the capacity of different chaotropic salts to support silica binding. Both NaI and NaCl could substitute for GITC to support similar binding of viral RNA to GM (Figures 2A and S1A). It should be noted that, in three of six combined nose and throat swabs, SARS-CoV-2 RNA was also detected in the absence of chaotropic salts (water sample), presumably as heat inactivation or chaotropic salts contained within the VTM (containing Hank’s balanced salt solution) may cause some viral particles to disassemble (Smyrlaki et al., 2020); RNase P was also detectable without major changes, potentially as lysed cells will release highly abundant RNase P RNA. As a ubiquitously available and inexpensive salt, we chose NaCl as the chaotropic salt for future experiments.

To improve viral particle solubilization from combined nose and throat swabs concomitant with inactivation (Patterson et al., 2020), we compared different detergents applied to the swabs. One hundred microliters of either 4% SDS, 4% Igepal, 4% Tween 20, 4% Triton X-100, or water were added to 100 μL of swab sample, and the GM protocol was followed afterwards. Neither detergent changed the detection sensitivity with a clear, reproducible trend (Figures 2B and S1B). Since SDS showed the lowest mean difference to QIAamp (ΔCt = −2.579 for N1 and ΔCt = −2.762 for N2) and has been shown to inactivate SARS-CoV-2 at concentrations as low as 0.5% (Patterson et al., 2020), we included it in our protocol.

Chaotropic salt-mediated nucleic acid precipitation and SPRI binding is improved in the presence of low dielectric constant solvents, such as isopropanol, (reviewed in Tan and Yiap, 2009). Consistently, our protocol greatly increased test sensitivity in the presence of 50% 2-propanol (Figures 2C and S1C). We also assessed the need for ethanol washes, which showed that at least one 70% ethanol wash should be applied to the GM to remove protein contamination, increasing sample purity and thus improving detection (Figures 2D and S1D).

One advantage of GM is its fast separation under low g-forces, enabling the use of cheap benchtop minicentrifuges or ultralow-cost devices (Bhamla et al., 2017; Li et al., 2020). We found that 7 mg of glass beads, corresponding to 10 μL of a 700 mg/mL GM solution, provided reproducible results and clean pelleting of the GM after 15 s of a pulse spin at 4,500 × *g* (Figures 2E and S1E).

Most silica-matrix SPRI protocols depend on a narrow range of pH of sample material for efficient binding (Tan and Yiap, 2009). Our GM protocol performed robustly over a pH range of 6.5–10 (Figures 2F and S1F). In combination, GM was able to reliably isolate SARS-CoV-2 (PHE isolate 1) diluted into VTM up to one plaque-forming unit per mL (PFU/mL; Figure 2G), a limit of detection comparable to that of QIAamp. Human RNase P has been suggested as an RT-PCR-positive control by the CDC. To assess the reliability of RNase P RNA isolation, we subjected serial dilutions of BEAS-2B bronchial epithelial cells resuspended in VTM and spiked with constant amounts of SARS-CoV-2 to the GM protocol. Figure 2H shows linear detection of RNase P. The detection of SARS-CoV-2 in these samples was affected, although still detectable, at dilutions of 12,500 cells or less.

Finally, we stress tested the GM protocol by inclusion of blood in combined nose and throat samples (20 μL in 80 μL of swab). Blood only marginally affected N2 detection, but not N1, and all samples remained positive (Figure S2A).

### Validating and pooling of homebrew with clinical samples

**Figure 3:**
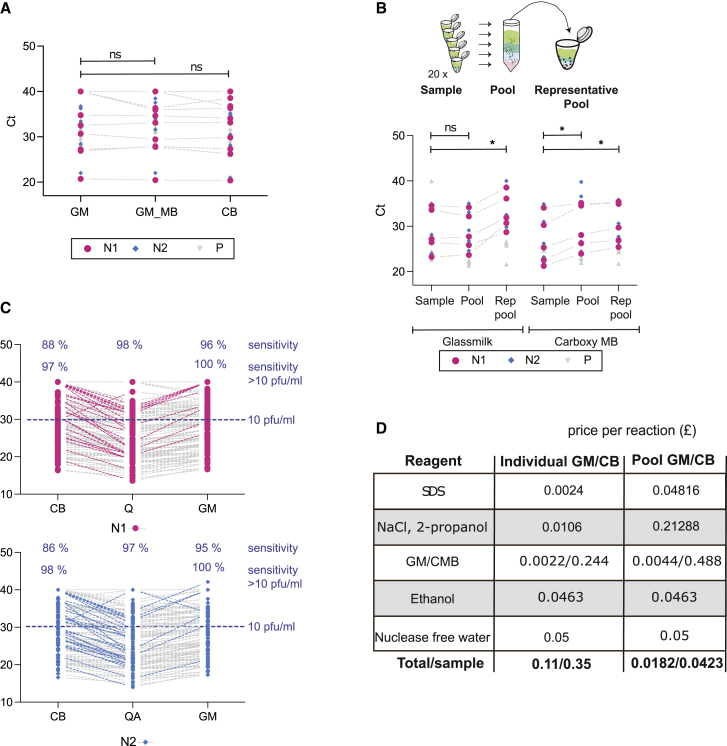
Validation and pooling capacity of the homebrew protocol (A) Comparison of GM, GM_MB, and CB. GM versus GM_MB (ns > 0.9999), GM versus CB (ns = 0.7807), Friedman test, two-tailed Dunn’s multiple comparisons test. (B) Pooling of samples does not decrease homebrew sensitivity. GM to GM pool (adjusted p = 0.6532) and GM versus GM rep pool (adjusted p = 0.019). Equally, CB versus CB pool (adjusted p = 0.011) and CB versus CB rep pool (adjusted p = 0.013). (C) Clinical validation of homebrew. Friedman test, two-tailed Dunn’s multiple comparisons test. (adjusted p < 0.0001 when comparing CB or GM to Q for N1 and N2). (D) Cost of GM and CB reagents based on UK list prices for individual samples and pooled samples.

Having established the individual components and limits of the GM protocol, we considered it crucial to reduce handling time and plastic usage to enable faster and cheaper high-throughput testing. High-throughput facilities employ commercial or open-source automated stations that rely on magnetic bead isolation (Lazaro-Perona et al., 2021). To this end, we prepared an extraction buffer that contained GM, 1.25M NaCl, and isopropanol (GM master buffer [GM_MB]) and compared this with the method employed thus far, where we added the components separately (GM). These two methods did not show statistical differences in the cycle threshold (Ct) values of the samples (Figure 3A; compare GM versus GM_MB). In addition, we prepared extraction buffers in which we exchanged GM for carboxy-coated magnetic beads (CB), which enable automation. Compared with GM, CB showed no statistical difference (Figure 3A).

Increased throughput for testing can also be achieved via sample pooling (Hogan et al., 2020). This typically consists of mixing an equal volume of different samples, taking a representative volume, and analyzing it in a single extraction and qRT-PCR (Lim et al., 2020). Employing column-based methods restricts pooling to small volumes with the consequent loss in sensitivity, particularly for samples with high Ct values. Others have suggested concentrating swabs prior to extraction and qRT-PCR (Sawicki et al., 2021). Although more sensitive, this increases processing time and costs for additional spin columns. As both GM_MB as well as CB_MB are based on solid matrices, either protocol allows scaling of the extraction for both manual and automated handling. To test this, we performed RNA extraction and qRT-PCR and compared Ct values obtained from five pools of samples that included one positive sample at different Ct values and 19 negative samples each (Figure 3C). One hundred microliters of a positive sample was either extracted individually (sample) or pooled with 19 negative samples (100 μL each). RNA from pooled samples was extracted in two different ways. One hundred microliters representative sample was taken from the pooled samples (rep pool) and extracted via the standard extraction protocol. Alternatively, we scaled SDS, NaCl, and isopropanol proportionally and extracted RNA from the entire sample volume (pool). It should be noted that the pooled extraction method only required GM for two reactions, i.e., 14 mg, making it a cost savings of 0.0396 per 20 samples (plus plastics) and significantly reducing the handling time. Figure 3B shows that, for GM, the “pool” Ct did not statistically significantly change compared with “sample” Ct, while the “rep pool” showed a significantly increased Ct for all pools. In the case of CB, both pool and rep pool showed an increase in Ct values of −2.996 and −3.789, respectively. Importantly, none of our pools was rendered negative, which shows the feasibility of using both GM and CB in pooled samples.

As a final comparison, we employed 100 clinical specimens and compared GM and automated CB with QIAamp (Figure 3C). Our data showed similar sensitivity for N1 primer probes between QIAamp and GM (98% versus 96%), while CB showed lower sensitivity (88%). To further show the reliability of this method, samples with >5 Ct difference to QIAamp were highlighted (filled lines; 9% GM; 32% GB). Considering swabs with a viral load corresponding to 10 PFU/mL (Ct = 30 for QIAamp based on Figure 2G), CB showed a sensitivity of 97% and GM 100% for N1 detection. N2 primer probes showed a slightly decreased sensitivity to that of N1: 86% (CB), 97% (QIAamp), and 95% (GM), which was increased to 98% (CB) and 100% (GM) in samples with a QIAamp Ct above 30. The approximate price per reaction of both GM and CB is summarized in Figure 3D, considering list prices of representative suppliers in the UK. Our estimate is that costs per extraction are reduced around 40-fold for individual samples and ∼400-fold for pooled samples.

## Discussion

In summary, we present a scalable, inexpensive, and simple method that uses reagents widely available in laboratories worldwide to detect viral genetic material from combined nose and throat swabs with high sensitivity.

Glassmilk-based isolation was initially described as a cheap alternative for plasmid preparations (Boyle and Lew, 1995; Vogelstein and Gillespie, 1979). However, with our current protocol, GM binds RNA preferentially, since recovery of a lentiviral plasmid was very poor (∼1%; Figure S2B), while that of small RNA (tRNA; ∼90 nt in length; Figure S2C) and cellular RNA (Figure S2D) was much greater.

Our ability to amplify RNase P in all nose and throat samples in our study (Figures 2, 3, and S1G–S1L) attests to the GM protocol’s capability of isolating cellular RNA. However, the quality of that RNA is low (Figure S2C), and rRNA appears degraded. We therefore note that, while it might be possible to use GM for cellular RNA isolation, the current protocol will require further adjustment. From preliminary experiments, we can say that cell lysis seems a key step in the process, since recovery of material was improved when cells were resuspended in PBS and lysed in a final 2% SDS concentration versus cells directly lysed in 4% SDS (Figure S2Ci). This was potentially also cell type specific, since two different cell lines (A549 and BEAS2B) rendered very different qualities (Figure S2Cii). Despite the suboptimal RNA quality, we were able to detect *GAPDH* and *IL8* mRNAs by qPCR with a limit of 250,000 cells per reaction, below which Ct values remained too high (Figure S2Ciii). This is in line with our data in Figure 2H, where we observed a loss in sensitivity for N1 and N2 detection when there was less RNA present in the samples. As SARS-CoV-2 detection decreases with lower amounts of cell input, we surmise that cellular RNA may act as carrier RNA, facilitating SARS-CoV-2 RNA binding to GM. Moreover, the DNA and RNA extracted employing homebrew GM did not contain DNase or RNase activity, since incubation of GM-isolated DNA or RNA with exogenous DNA and RNA did not cause observable degradation (Figure S2D). Thus, homebrew GM has the potential of being used to perform cellular RNA extractions, provided appropriate cellular lysis conditions.

Multiple novel approaches have been developed to evaluate SARS-CoV-2 infection (Eftekhari et al., 2021; Kevadiya et al., 2021). Different test types (e.g., antigen tests and serological tests) have their place according to needs and resources; however, qRT-PCR remains the most sensitive method and the gold standard to which all tests compare. We believe that our method is of value currently, as cases continue to soar in many parts of the world. The current pandemic has taught us that future zoonotic transmission is highly likely to cause outbreaks. Cheaper and simpler viral detection methods will therefore be continuously required. We thus believe that the method presented here has long-standing potential in viral disease control.

### Limitations of the study

We acknowledge that our study comes with limitations. Our experiments were performed on swabs that had been tested for the presence of SARS-CoV-2 and were subsequently frozen; the influence of freeze-thawing material cannot be measured in our study. In order to make our protocol useful for others, we introduced a first step of heat inactivation of swab material, as many laboratories face the problem of dealing with non-inactivated material. It is possible that the heat inactivation allows better performance of the master buffer made with water—and hence addition of detergents does not seem to strongly influence Ct values (Figures 2B and S1B). In addition, we compared GM with a similar SPRI matrix by comparing it with QIAamp only, as QIAamp is recommended by the CDC and we have not established a further comparison to other commercial SPRI silica-based systems. Finally, we have used ANOVA statistics to compare Ct values for scientific convention; however, for diagnostic purposes, we are only interested in gain or loss of sensitivity (i.e., positive or negative sample). A more appropriate approach could be the reporting of mean delta Ct values, which we used to validate the reagents used in our method (Table S1). As in our previous work, we recommend considering the following parameters to establish a sample as positive or negative (Table 1). In both inconclusive (unclear viral amplification) and void (not enough material, as RNase p values too high) results, a new sample from the same individual must be tested as soon as possible.

**Table 1:** Parameters suggested to establish positive or negative in samples

Result	Positive	Negative	Inconclusive	Void
N1	<36, regardless of N2 amplification	undetermined	≥36 and N2 negative	–
N2	<36, regardless of N1 amplification	undetermined	≥36 and N1 negative	–
RNase P	<35	<35	<35	≥35

## STAR★Methods

### Key resources table

**Table:** 

REAGENT or RESOURCE	SOURCE	IDENTIFIER
**Chemicals, peptides, and recombinant proteins**		

Silicon dioxide 325 mesh	Sigma/Merck	Cat# 342890
Carboxylate modified magnetic SpeedBeads	Sigma/Merck	Cat# GE45152105050250
SDS (500g)	Sigma/Merck	Cat# L3771-500G
NaCl (500g)	Sigma/Merck	Cat# S3014-500G
Isopropanol (2.5L)	Fisher scientific	Cat# BP2618-212 2.5L
Nuclease Free Water (500mL)	Fisher Scientific	Cat# AM9930
Triton X-100	Sigma/Merck	Cat# X100-500mL
Tween-20	VWR	Cat# 663684B
Igepal	Generon	Cat# NDB0385

**Critical commercial assays**		

Qiagen QIAmp Viral RNA Mini Kit (250)	Qiagen	Cat# 52906
TaqMan Fast Virus 1-Step Master Mix	Thermo Fisher Scientific	Cat# 4444434
2019-nCov CDC EUA Kit	Integrated DNA Technologies	Cat# 10006770
RevertAid H Minus Reverse Transcriptase	Thermo Fisher Scientific	Cat# EP0452
Random Hexamers	Thermo Fisher Scientific	Cat# SO142
Deoxynucleotide set, 100 mM, 0.25 mL each	SIGMA	Cat# DNTP100-1KT
Luna® Universal Probe qPCR Master Mix	New England Biolabs	Cat# M3004E

**Deposited data**		

Data sets generated in this paper		https://data.mendeley.com/datasets/b2mscbnhmg/2

**Experimental models: Organisms/strains**		

SARS-CoV-2 PHE isolate England02	Public Health England	N/A

**Oligonucleotides**		

2019-nCoV_N1 Forward Primer: GAC CCC AAA ATC AGC GAA AT		2019-nCoV_N1-F 500nM
2019-nCoV_N1 Reverse Primer: TCT GGT TAC TGC CAG TTG AAT CTG		2019-nCoV_N1-R 500nM
2019-nCoV_N1 Probe: FAM-ACC CCG CAT TAC GTT TGG TGG ACC-BHQ1		2019-nCoV_N1-P 150nM
2019-nCoV_N2 Forward Primer: TTA CAA ACA TTG GCC GCA AA		2019-nCoV_N2-F 500nM
2019-nCoV_N2 Reverse Primer: GCG CGA CAT TCC GAA GAA		2019-nCoV_N2-R 500nM
2019-nCoV_N2 Probe: FAM-ACA ATT TGC CCC CAG CGC TTC AG-BHQ1		2019-nCoV_N2-P 150nM
RNAse P Forward Primer: AGA TTT GGA CCT GCG AGC G		RP-F 500nM
RNAse P Reverse Primer: GAG CGG CTG TCT CCA CAA GT		RP-R 500nM
RNAse P Probe: FAM – TTC TGA CCT GAA GGC TCT GCG CG – BHQ-1		RP-P 150nM
GAPDH	Primer Design	
IL6, Assay ID Hs00174131_m1, S	Thermo Fisher Scientific	4331182

**Software and algorithms**		

GraphPad Prism		https://www.graphpad.com/scientific-software/prism/
QuantStudio Software		QS5 (v.1.5.2) https://www.thermofisher.com/uk/en/home/global/forms/life-science/quantstudio-3-5-software.html< QS7 Flex (v1.7.1): https://www.thermofisher.com/uk/en/home/global/forms/life-science/quantstudio-6-7-flex-software.html

### Resource availability

#### Lead contact

Further information and requests for resources and reagents should be directed to and will be fulfilled by the lead contact, Rocio T. Martinez-Nunez rocio.martinez_nunez@kcl.ac.uk.

#### Materials availability

This study did not generate new unique reagents.

### Experimental model and subject details

Samples for this study were provided under KCL TEST (KCL Ethics Ref: 21150); and Service Delivery for King’s College Hospital. All samples were combined nose and throat swabs having already been tested at King’s College Hospital or KCL TEST as part of a potential service development. All samples were anonymised and assessed after being diagnosed as SARS-CoV-2 positive by the hospital or negative by KCL TEST. Samples were chosen based on Ct values in order to represent a broad range of values. Positive swabs were inactivated in a Category 3 facility employing 90°C 10 mins in a dry bead bath (Lista et al., 2021).

### Method details

#### Glass milk preparation

*Important note: work under fume hood.* Suspend silicon dioxide 325 mesh (Sigma 342890) in 40mL 10% HCl from a 37% HCl stock diluted in water in a 50mL tube, agitate suspension for 4 hrs on a tube roller and centrifuge at 2000 g. Remove supernatant and resuspend the silica pellet with 40mL water. Centrifuge and repeat the water wash five times (a total of six washes). Make sure the silica is fully resuspended after each wash so residual HCl is not ‘trapped’ in the pellet mass. After the final wash, weigh the pellet and resuspend in water at 700 mg/mL. The pH of the silica should be between 7 and 8.

#### VTM preparation

Viral transport medium was prepared as per CDC instructions (https://www.cdc.gov/coronavirus/2019-ncov/downloads/Viral-Transport-Medium.pdf), i.e. 2% FBS 100μg/mL Gentamicin 0.5 μg/mL Amphotericin B in Hanks Balanced Salt Solution (HBSS).

#### RNA extraction

##### Standard protocol

Combined nose and throat swab samples were heat-inactivated at 90°C for 10 min in a Cat-3 facility. Swabs that did not contain enough volume for all comparisons were topped up with negative combined nose and throat swabs in viral transport medium. In a 1.5 mL tube, 100μL of sample were mixed with 100μL of 4% SDS. 610 μL of glassmilk extraction buffer (GM_MB) (200 μL 1.25 M NaCl, 400 μL 2-propanol and 10μL of GM) were added to each sample, the mixture was inverted, vortexed and incubated at room temperature for 5 minutes. The sample was then centrifuged for 15 seconds in a bench-top microfuge at 4600 x g and the supernatant poured off. The silica pellet was resuspended in 500 μL of 70% ethanol before being centrifuged for 15 seconds at 4600 x g (wash 1). The supernatant was poured off and the silica pellet was resuspended again in 500 μL of 70% ethanol and centrifuged for 15 seconds at 4600 x g (wash 2). After another spin at 4600 x g (15 sec), all remaining ethanol in the supernatant was carefully removed with a pipette and the silica pellet was air-dried at 65°C for 5 minutes. Finally, RNA was eluted by resuspending the silica pellet in 50μL of nuclease-free water. The tube was centrifuged for 15 seconds and the supernatant containing RNA was transferred to a fresh 1.5mL tube.

##### Adaptation to magnetic beads

All steps as above, except that the GM_MB contained 10 μL 50 mg/mL carboxylate modified magnetic SpeedBeads (Merck GE45152105050250). To collect beads manually, we employed a magnetic rack (Invitrogen, DynaMag™-2 Magnet, 12321D). Beads were dried for 5 minutes at room temperature.

##### Adaptations for individual figures

Figure 2A: testing different salts. The same protocol as described above was used, except that instead of GM-EB, we added its components separately to allow assessment of individual chaotropic salts: 100μL of sample was mixed with 100μL water to which 200 μL of either 1.25M NaCl, 1.25M NaI, 1.25M GITC or water were added. 10μL of GM [700 mg/mL] and 400μL of isopropanol was added before incubating the mixture at room temperature for 5 minutes.

Figure 2B: testing different detergents. We mixed 100 μL of combined nose and throat swabs with 100 μL of either 4% SDS, 4% Igepal, 4% Tween, 4% Triton-X100 or water, before 200 μL 1.25 M NaCl, isopropanol and 10μL of GM [700 mg/mL] were added and the standard protocol followed thereafter.

Figure 2F: assessment of pH ranges. 100 μL swab material was inactivated for 10 min at 90 °C before 100 μL of 4% SDS were added. NaCl solutions were buffered with hydrochloric acid to different pH so that mixing with the swab would yield the pH indicated in the figure: NaCl buffered to pH1.5 results in pH 4.5 after mixing; NaCl buffered to pH3, 6.75, 10.75 and 11 resulted in swabs with pH 6.5, 6.75 8.1 and 9.8, respectively, after mixing.

Figure 3B Pooling: 100 μL heat-inactivated swab material (10 min at 90 °C) from 20 different samples were mixed in a 50 mL tube. To this mixture, 2 mL of 4% SDS, 4 mL of 1.25 M NaCl, 8 mL of isopropanol and 20 μL of GM were added. Samples were mixed and spun, washed twice with 1 mL 70% ethanol in a 1.5 mL tube and the GM pellet was air dried at 65° C for 5 min prior to resuspending in 50 μL of nuclease free water.

Figure 3C: RNA extraction for CB samples was performed using a KingFisher Flex automated station.

RNA extraction with QIAamp Viral RNA Mini kit (QIAGEN) was conducted as per the manufacturer’s instructions. To allow comparison with the above method, 100 μL of heat-inactivated sample was used as input material and 50 μL of nuclease-free water was used to elute RNA from the column.

Plasmid DNA purified in Figure S2B was that of pLVTHM_shRNA control (Martinez-Nunez et al., 2017).

#### RT-qPCR

RT-qPCR reactions were carried out using TaqMan Fast Virus 1-Step Master Mix (ThermoFisher Scientific, 4444434). Primer-probe sets for used SARS-CoV-2 detection were the CDC-recommended sets N1, N2 and RNaseP Emergency Use Authorisation kit (Integrated DNA Technologies, 2019-nCov CDC EUA Kit, 10006770). RT-qPCR reactions containing 5 μL master mix, 1.5 μL pre-mixed primer-probe, 8.5 μL water and 5 μL purified RNA were run on a QuantStudio 5 (Applied Biosystems/ThermoFisher Scientific) using the “Fast” cycling mode. Figure S2 experiments were run on a QuantStudio 7 Flex (Applied Biosystems/ThermoFisher Scientific). The cycling conditions used were 50° C 5 mins, 95° C 20 sec, and 45 cycles of denaturation (95° C, 3 sec) and annealing/extension (60° C, 30 sec). RT-qPCR reactions were performed in duplicate for all samples, except for those used in Figure 3B to resemble a testing setting and done in singlets. Undetermined samples were set at a Ct of 40 for statistical and representation purposes, except in the GM titration figure (Figure 2F) where we considered values above 40 to stress-test the system.

RT-qPCR for cellular RNAs in Figure S2Ciii was done in two steps. 150 ng of RNA were reverse transcribed with RevertAid H Minus Reverse Transcriptase (ThermoFisher Scientific) using 100pmol random hexamers in a final volume of 10 μL (25° C, 10 min; 42° C, 60 min; 70° C, 10 min). 1:5 diluted complementary DNA (cDNA) was amplified by qPCR using GAPDH (Primer Design) and IL6 (ThermoFisher Scientific) primers and the Luna® Universal Probe qPCR Master Mix (New England Biolabs). 1 μL of diluted cDNA was mixed with 5 μL master mix buffer, 0.25 μL primer-probe mix and 3.75 μL of nuclease free water in a final reaction of 10 μL. Cycling parameters (in fast mode) were 95°C 60 sec, and 45 cycles of denaturation (95°C, 15 sec) and annealing/extension (60°C, 30 sec).

### Quantification and statistical analyses

Statistics were performed on the Ct values obtained by amplification using the N1 primer-probe sets in Figures 2 and 3 and N2 and RNAseP in Figure S1. All datasets were initially assessed for normality distribution using a Kolmogorov-Smirnov test. Parametric data were compared using a one-way ANOVA with a multiple correction Dunnett’s test. Non-parametric data were analysed using a Friedman test with Dunn’s multiple comparison test. Paired t-tests were employed in Figure S2A. Reported *P*-values are p-adjusted values from the multiple comparison tests. *P*-value was deemed significant when p-adjusted was < 0.05. ∗: *P*-adjusted < 0.05; ∗∗ *P*-adjusted < 0.01; ∗∗∗: *P*-adjusted <0.001; ∗∗∗∗: *P*-adjusted < 0.0001.

## Data Availability

• RT-qPCR data have been deposited at Mendeley at https://data.mendeley.com/datasets/b2mscbnhmg/2, and are publicly available as of the date of publication. The DOI is listed in the key resources table. • This paper does not report original code. • Any additional information required to reanalyze the data reported in this paper is available from the lead contact upon request. • According to UK research councils’ Common Principles on Data Policy, all data supporting this study will be openly available at https://data.mendeley.com/datasets/b2mscbnhmg/2. • According to Wellcome Trust’s Policy on data, software, and materials management and sharing, all data supporting this study will be openly available at https://data.mendeley.com/datasets/b2mscbnhmg/2.

## Supplemental information

Document S1. Figures S1 and S2

Table S1. Ct values of Figures 2 and 3.

Document S2. Article plus supplemental information
