# Supplementary material for: Homebrew: An economical and sensitive glassmilk-based nucleic-acid extraction method for SARS-CoV-2 diagnostics
Source: Cell Rep Methods. 2022 Mar 3;2(3):100186. doi: 10.1016/j.crmeth.2022.100186 (PMC8890991; doi:10.1016/j.crmeth.2022.100186)
Supplement: Document S1. Figures S1 and S2 [file mmc1.pdf]

Cell Reports Methods, Volume *2*

Supplemental information

Homebrew: An economical and sensitive glassmilk-based nucleic-acid extraction method for SARS-CoV-2 diagnostics

Robert Page, Edward Scourfield, Mattia Ficarelli, Stuart W. McKellar, Kwok Leung Lee, Thomas J.A. Maguire, Clement Bouton, Maria Jose Lista, Stuart J.D. Neil, Michael H. Malim, Mark Zuckerman, Hannah E. Mischo, and Rocio T. Martinez-Nunez

Figure S1: N2 (A-F) and RNAseP (G-L) values corresponding to Figure 2

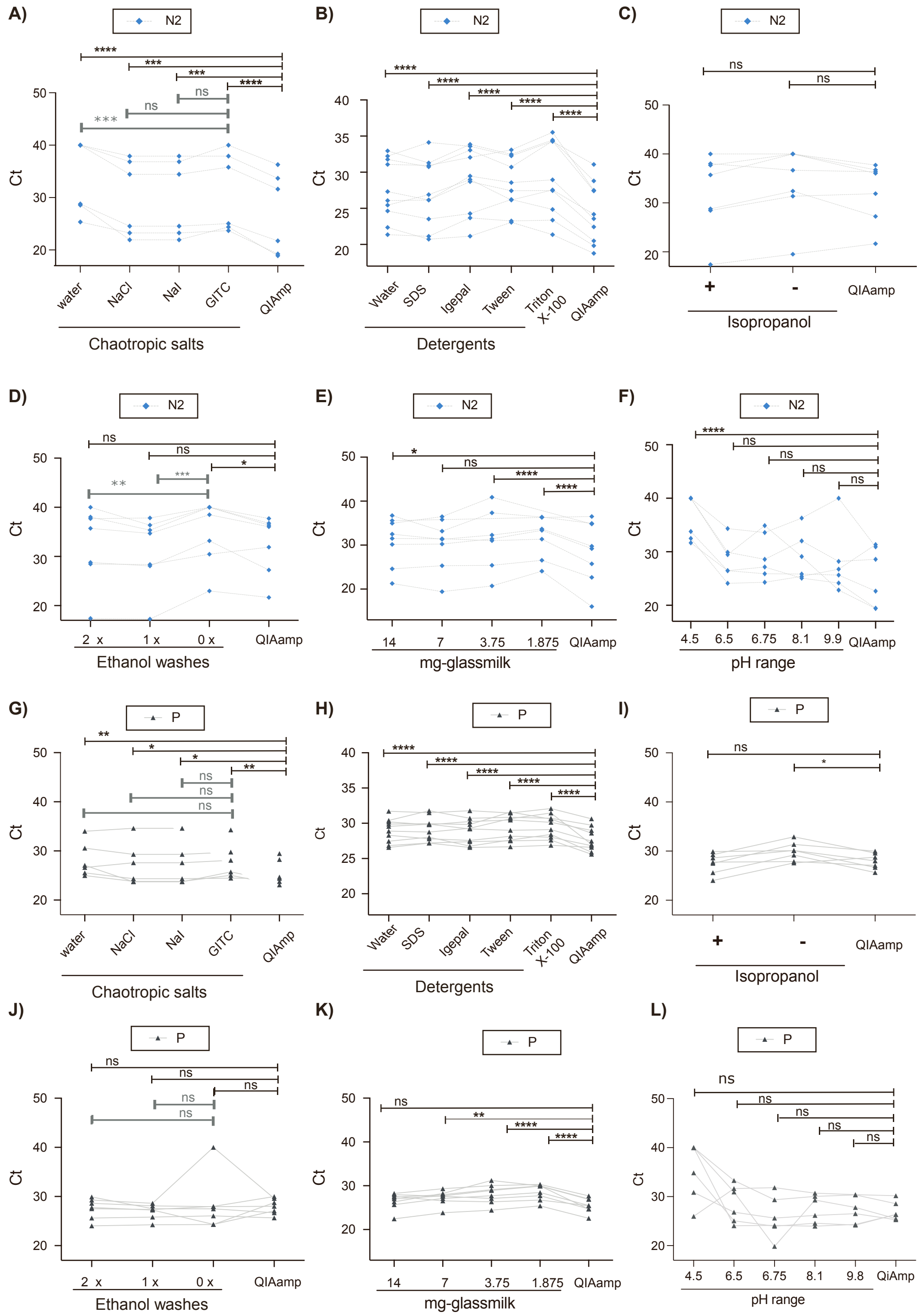

Figure S1: N2 (A-F) and RNAseP (G-L) values corresponding to Figure 2. A and G). Chaotropic salt titration: Viral RNA extracted from six combined nose and throat swabs using different chaotropic salts, 2-propanol and 7 mg GM, shows in a one-way ANOVA test significant increase in Ct values compared to the bench-mark QIAamp viral RNA mini kit. B) and H). Detergent titration: Addition of different detergents prior to RNA extraction from ten combined nose and throat swabs, shows a significant difference to QIAamp (adjusted *P*-adj one-way ANOVA of < 0.0001. All detergents but Tween-20 have been shown to inactivate SARS-CoV-2 at 1 % (Patterson et.al., 2020). C) and I). Isopropanol effect on GM binding. Friendman's test with Dunn's multiple comparison showed non-significant differences by adjusted *P*-adj for N2 but a loss in sensitivity in detecting RNAseP as per ANOVA analysis. D) and J). Matrix wash with Ethanol increases sensitivity. One-way ANOVA test shows no significant differences to the QIAamp viral RNA mini kit (D) and Friendman's test with Dunn's multiple comparison (J) showed loss of detection of RNAseP for one sample. E) and K). GM quantities. ANOVA test with Dunnett's multiple comparison test vs QIAamp showed no difference in N2 detection using 7mg GM vs QIAamp and *P-adj* 0.0021 for RNAseP detection (mean difference -1.729). F) and L). Effect of pH of the RNA-extraction buffer on detection of viral RNA from 6 combined nose and throat swabs compared to QIAamp. Only pH 4.5 showed a significant difference vs QIAamp for N2 detection (F) while detection of RNAseP (L) appeared not affected.

Figure S2: Effect of blood on homebrew GM and versatility of the method.

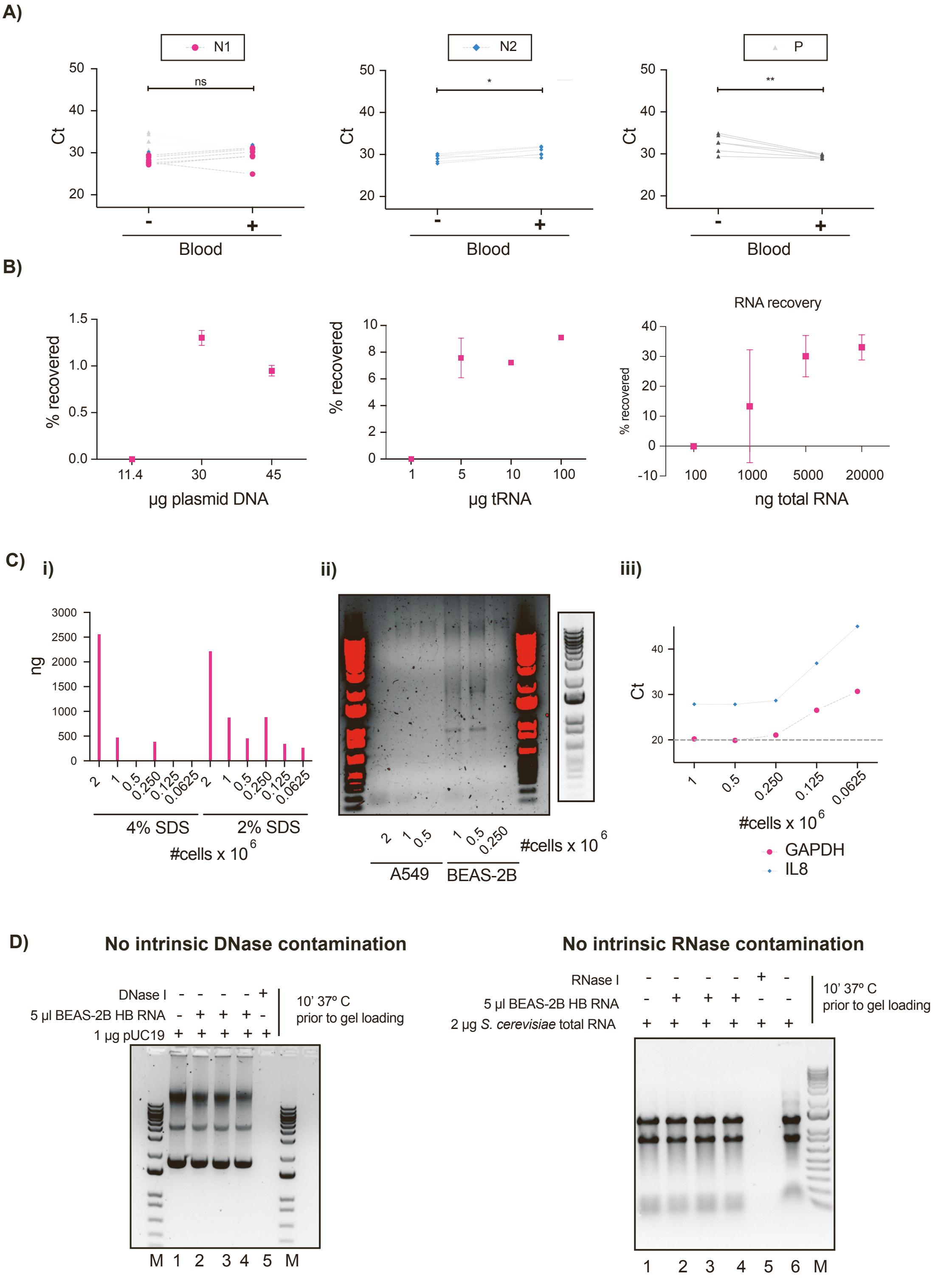

Figure S2: Effect of blood on homebrew GM and versatility of the method. A) Effect of blood in sensitivity of detection of SARS-CoV-2. 6 negative combined nose and throat swabs were spiked with SARS-CoV-2 and heat inactivated, and blood was added (or not). Sensitivity of N1 detection appeared not affected, with N2 increasing in Ct marginally ($P = 0.0136$). Paired t-tests were employed. More RNAseP was detected when swabs were spiked with blood, as expected. B) Recovery of plasmid DNA (left), small RNA (middle, tRNA, ~90nt) and cellular RNA (right) by homebrew GM. C) Recovery of cellular RNA from cultured cells depending on lysis conditions (i) as measured by Qubit, RNA integrity (ii) and Ct values of GAPDH and IL8 in 150ng of RNA isolated from different cell numbers (iii). D) DNAse (left) and RNAse (right) assessment of GM-isolated extracts. Plasmid DNA (left) or cellular RNA (right) were incubated with DNA or RNA extracted employing GM homebrew.
